# Hepatitis E Virus in Croatian Liver Transplant Recipients: Seroprevalence and One-Year Post-Transplant Surveillance from a Combined Cohort Study (2019–2022)

**DOI:** 10.3390/microorganisms13092063

**Published:** 2025-09-04

**Authors:** Petra Dinjar Kujundžić, Tatjana Vilibić-Čavlek, Tomislav Kelava, Alan Ayoub, Jelena Prpić, Lorena Jemeršić, Adriana Vince, Anna Mrzljak

**Affiliations:** 1 Department of Gastroenterology, Hepatology and Clinical Nutrition, University Hospital Dubrava, 10000 Zagreb, Croatia; 2 Department of Virology, Croatian Institute of Public Health, 10000 Zagreb, Croatia; tatjana.vilibic-cavlek@hzjz.hr; 3 School of Medicine, University of Zagreb, 10000 Zagreb, Croatia; tomislav.kelava@mef.hr (T.K.); avince@bfm.hr (A.V.); 4 Department of Emergency Medicine, University Hospital Dubrava, 10000 Zagreb, Croatia; alan.ayoub555@gmail.com; 5 Department of Virology, Croatian Veterinary Institute, 10000 Zagreb, Croatia; jelena.prpic.jp@gmail.com (J.P.); jemersic@veinst.hr (L.J.); 6 Department of Virology, University Hospital for Infectious Diseases, 10000 Zagreb, Croatia; 7 Department of Hepatology, University Hospital Center Zagreb, 10000 Zagreb, Croatia

**Keywords:** epidemiology of HEV infection, chronic HEV infection, liver transplantation

## Abstract

Hepatitis E virus (HEV) is a significant yet understudied cause of viral hepatitis among liver transplant (LT) recipients in Central and South-eastern Europe. We conducted a combined cross-sectional and prospective cohort study to determine the seroprevalence, incidence, and risk factors of HEV infection in Croatian LT recipients. A total of 766 adult LT recipients were analyzed for anti-HEV IgG and HEV RNA. Additionally, 152 patients listed for LT were followed prospectively with sampling before and up to 12 months after transplantation. Anti-HEV IgG seroprevalence in the cross-sectional cohort was 19.8%, with no active infections detected via HEV RNA. In the prospective cohort, baseline seroprevalence was 20.4%, declining post-transplant, while 3.9% of initially seronegative patients seroconverted without detectable HEV RNA. Older age and non-tertiary level of education were associated with seropositivity, while dietary and animal contact factors were not. These findings indicate a moderately high level of prior HEV exposure among Croatian LT recipients, comparable to some European regions, but a low incidence of post-transplant infection and no evidence of chronic HEV infection. Our results suggest that despite frequent exposure, clinically significant HEV infection is uncommon in this immunosuppressed population.

## 1. Introduction

Hepatitis E virus (HEV) is currently the predominant cause of acute viral hepatitis in humans. In particular, members of the species *Paslahepevirus balayani*, including HEV-1 to HEV-4, are recognized as the causes of major public healthcare concerns. While G1 and G2 were initially considered endemic only in developing countries with poor sanitation and are primarily transmitted via contaminated water, genotypes G3 and G4 are identified as major culprits in sporadic cases across industrialized nations showing zoonotic potential [1]. In Europe, HEV-3 is the predominant genotype, transmitted mainly via consumption of undercooked pork or direct animal contact with infected animals [2]. Importantly, it is also recognized as the cause of chronic hepatitis in immunocompromised individuals, including organ transplant recipients [3].

Liver transplant (LT) recipients are at particular risk of HEV infection due to lifelong immunosuppressive therapy and frequent exposure to blood products and donor organs. Chronic HEV infection in these subjects can lead to rapid progression of liver fibrosis and graft loss [3]. Although European data suggest varying levels of HEV seroprevalence among transplant populations, from as low as 1–3% in Greece and the Netherlands to over 25% in particular areas of Germany and France, regional and methodological differences limit generalizability [4,5,6,7].

In Croatia, HEV infection is an emerging public health concern. Studies confirm its endemicity in domestic pigs and wild boars, indicating a permanent risk of spread to humans [8,9]. Furthermore, previous reports have shown moderate to high HEV IgG seroprevalence in several subpopulations, including blood donors (21.5%), hemodialysis patients (27.9%), and individuals with chronic liver disease (15.1%) [10]. However, data on the post-transplant population have been limited to small cohorts [11].

We conducted a hybrid study at Croatia’s national referral center comprising a cross-sectional survey and a prospective cohort. The cross-sectional component estimated anti-HEV IgG seroprevalence and the point prevalence of acute infection at sampling (HEV RNA-positive). The prospective component analyzed 12-month post-transplant incidence of HEV infection (RNA-positive) and anti-HEV IgG kinetics. Together, these complementary designs provide a comprehensive epidemiologic profile of HEV in Croatian LT recipients.

Accordingly, our objectives were to (1) estimate anti-HEV IgG seroprevalence; (2) assess the point prevalence of RNA-confirmed acute HEV infections at the cross-sectional visit; (3) determine the 12-month post-transplant incidence of HEV RNA-positive infections; (4) evaluate the occurrence of chronic HEV infections; (5) identify demographic, clinical, and lifestyle factors associated with HEV exposure; and (6) analyze anti-HEV IgG dynamics over time.

## 2. Methods

### 2.1. Study Design

We conducted an observational hybrid study with two complementary components within a single national center. The cross-sectional survey estimated the cumulative burden of prior exposure and assessed the point prevalence of acute HEV infection at the study visit, defined strictly as HEV RNA positivity. The prospective cohort quantified the 12-month post-transplant incidence of RNA-confirmed HEV infection and characterized anti-HEV IgG kinetics (including seroconversion) under current practice. Seroconversion without positive HEV RNA was treated as a serologic outcome and not counted as a new infection. Both components were conducted at Merkur University Hospital, Zagreb, the national reference center for liver and multivisceral transplantation, which performed >90% of liver transplants in Croatia during the study period.

### 2.2. Study Period and Participants

Eligibility. All patients aged ≥18 years who underwent liver transplantation (LT) at Merkur University Hospital were eligible; no exclusion criteria were applied.

Cross-sectional cohort: We included 766 consecutive adult LT recipients transplanted 1994–2019, recruited during routine outpatient visits January–December 2022. At the visit, each participant provided a venous blood sample and completed a single structured questionnaire on putative HEV exposures. Specimens were stored and later batch-tested for anti-HEV IgG and HEV RNA.

Prospective cohort: We enrolled 152 consecutive patients listed for LT (September 2019–December 2022) who underwent transplantation and were scheduled for blood sampling at baseline (pre-LT) and at approximately 6 and 12 months post-LT. Follow-up occurred during scheduled transplant clinic visits and was supplemented by review of clinical records; blood from each time point was stored and later tested using the same assays as in the cross-sectional component. In this cohort, inclusion required completion of both follow-up visits, yielding an incidence denominator of 152; the exposure questionnaire was administered once per participant at either the 6- or 12-month visit, according to scheduling.

Outcomes: In the cross-sectional component, outcomes were (i) anti-HEV IgG seroprevalence and (ii) the point prevalence of acute HEV infection at the study visit, defined strictly as HEV RNA positivity. In the prospective component, the primary outcome was the 12-month incidence of RNA-positive HEV infection, and secondary outcomes were anti-HEV IgG kinetics (including seroconversion). Chronic HEV infection was defined a priori as persistent HEV RNA positivity for more than three months in an immunocompromised host.

Patient characteristics and potential exposures: We recorded demographics (age, sex); transplant-related variables (calendar year of LT and time since LT at sampling); immunosuppression (monotherapy, dual, or triple therapy; calcineurin inhibitor category: tacrolimus or cyclosporine); clinical factors (etiology of liver disease and comorbidities); and lifestyle/environmental factors (consumption of pork/offal, undercooked meat, animal contact, rural residence, blood transfusions, and travel). Laboratory variables included anti-HEV IgG indices, HEV RNA results, and liver biochemistry.

### 2.3. Laboratory Testing

For each sampling, 2 mL of venous blood was drawn. The blood was centrifuged, and the serum was separated and stored at −20 °C until testing. Serological testing for anti-HEV IgG antibodies was performed using a commercial enzyme immunoassay (recomWell HEV IgG, Mikrogen GmbH, Neuried, Germany). Samples that tested reactive in the initial screening were further confirmed using an immunoblot (IB) test (recomBlot IgG anti-HEV, Mikrogen GmbH, Neuried, Germany), using highly purified recombinant HEV antigens: O2N genotype 1/3, O2C genotype 1/3, O2M genotype 1, and O3 genotype 1/3. According to the manufacturer’s specifications, the IB has a diagnostic sensitivity of 96.6% and a specificity of 97.1%.

In this study, automated extraction of viral RNA was used to detect HEV infection in liver transplant recipients. Total RNA was extracted from whole blood samples using the iPrep™ PureLink™ Total RNA Kit (Invitrogen, Life Technologies, Carlsbad, CA, USA), based on magnetic separation combined with ChargeSwitch^®^ technology. Samples were lysed under denaturing conditions to inactivate RNases, incubated, and centrifuged. The supernatant was then processed using the iPrep™ Purification Instrument. Final RNA volume was 50 µL, stored at −80 °C. HEV RNA detection was performed via reverse transcription-polymerase chain reaction (RT-qPCR) targeting the ORF3 gene, using 3 µL RNA in a 20 µL reaction with the 1Step RT-PCR Probe ROX L Kit (highQu). Primers and probes followed the protocol by Jothikumar et al. (2006) [12]. Amplification was performed on a Stratagene Mx3005P (Agilent Technologies, Santa Clara, CA, USA). Samples with Ct < 40 were considered positive. Positive and negative controls were included in each run to validate the results. This method ensures sensitive detection of HEV RNA, essential in immunocompromised patients where serology may be unreliable.

### 2.4. Data Collection

Demographic and clinical data were collected for each participant, including age, gender, date of transplantation, geographic region of residence, underlying liver disease, and details of immunosuppressive therapy. During each visit, standard laboratory parameters were assessed, including white blood cell count, lymphocyte count, platelet count, aspartate aminotransferase (AST), alanine aminotransferase (ALT), C-reactive protein (CRP), and blood levels of calcineurin inhibitors (CNIs). These analyses were performed using validated protocols in the certified clinical laboratory.

Participants completed a questionnaire on potential HEV risk factors, including education, household size, sanitation, domestic animals, diet, home-processed meat consumption, animal exposure, blood transfusion history, and travel abroad.

### 2.5. Study Size

The study size was determined based on the number of eligible patients during the study period. For the cross-sectional cohort (n = 766), the precision for a prevalence estimate of 20% was ±3% at the 95% confidence level. For the prospective cohort (n = 150), the study could detect an incidence of 2–3% with 95% confidence intervals of 0.7–5.7% and 1.4–7.6%, respectively.

### 2.6. Statistical Analysis

Categorical variables are presented as n (%) and continuous variables as median (interquartile range; IQR). Percentages were calculated using available case denominators; missing values were not imputed. Hypothesis tests excluded missing observations, and multivariable analyses used complete-case data. Group comparisons used Pearson’s χ^2^ (or Fisher’s exact when any expected cell <5) and the Mann–Whitney U test. Overall anti-HEV IgG seroprevalence was estimated with 95% CIs (Wilson). Determinants of seropositivity were assessed with multivariable logistic regression; variables with *p* < 0.20 in preliminary bivariate comparisons were entered into the model. Assumptions were checked (logit linearity for continuous predictors; multicollinearity via VIF). The results are reported as odds ratios (Exp[B]) with 95% CIs, alongside coefficients (B), standard errors, and Wald χ^2^. Anti-HEV IgG kinetics (including seroconversion) were summarized descriptively and not treated as incident infection. All tests were two-sided (α = 0.05); analyses were performed in SPSS v19.0 (IBM Corporation, Armonk, NY, USA).

## 3. Results

### 3.1. Cross-Sectional Cohort

This cohort included 766 liver transplant recipients who underwent transplantation between 1994 and 2019. The majority of patients were male 68.6% (526/766), with a median age of 56 years (50.5–62.5) at the time of transplantation and 60 years at the time of sampling. Anti-HEV IgG antibodies were detected in 19.8% (152/766) of patients, while HEV RNA was not detected in any of the analyzed samples.

The predominant etiology of liver disease was alcohol-related liver disease (47.0% 360/766), followed by primary liver tumors (25.0%; 199/766), viral hepatitis (17.1% 131/766), and autoimmune liver diseases (9.9%; 76/766). Most patients (77.8%; 596/766) were receiving dual immunosuppressive therapy, primarily a calcineurin inhibitor (53.4%; 409/743 tacrolimus, 42.4%; 325/743 cyclosporine) in combination with an antimetabolite. Routine laboratory parameters showed stable post-transplant values.

Sociodemographic data showed that 61.2% (445/743) had secondary education, and 52.1% (387/743) lived in urban areas. Processed meat consumption was reported by 63.8% (489/766), of which 22.3% (171/766) products were homemade. Occupational animal exposure was noted in 14.8% (113/766), while 24.3% (186/766) owned a farm. Most used public water supplies (86,9%; 602/693) and public sanitation (58,2%; 430/738). Blood transfusions were reported by 65.4% (499/743), and 27.8% (213/766) reported international travel.

When comparing liver transplant recipients by anti-HEV IgG serostatus, seropositive patients were older at the time of sampling (median 61 vs. 59 years; *p* = 0.023). Education level also differed significantly (*p* = 0.017), with the lowest seroprevalence among those with tertiary education (4.6%) and higher proportions in those with secondary education or no formal education. No significant differences were observed by gender, residence, animal contact, dietary habits, animal exposure, water source, sanitation type, or recent travel abroad. Seroprevalence varied regionally, with the highest recorded in Slavonia (37.8%) and the lowest in Istria (7.4%), though these differences were not statistically significant (Table 1).

Multivariable logistic regression confirmed that older age (OR = 1.03; 95% CI 1.01–1.05; *p* = 0.009) was independently associated with anti-HEV IgG seropositivity, while tertiary education was protective (OR = 0.33; 95% CI 0.15–0.74; *p* = 0.007). No significant independent associations were found for household size, vegetarian diet, occupational exposure to animals, or history of chronic hepatitis B or C (Table 2).

### 3.2. Prospective Cohort

This group included 152 subjects with end-stage liver disease listed for LT. HEV RNA was undetectable in all samples at all three time points. By the RNA-based definition, the 12-month incidence of HEV infection was 0/152 (0.0%; 95% CI 0.0–2.4%). The six post-LT seroconversions were analyzed as serologic kinetics only and were not classified as previous infection. The majority were male (66.0%; 100/152), with a median age of 59.5 years. The most common underlying cause of chronic liver disease was alcohol-related liver disease 36.8% (56/152). Tacrolimus was the most used calcineurin inhibitor (69.7% 106/152).

Demographic data showed that patients were almost equally distributed between rural and urban areas (49%; 75/152 vs. 51%; 77/152). Most participants lived in households with three to four members (59.0%; 90/152), while smaller households with fewer than two members and larger ones with more than five members each accounted for 20.0% (31/152) of the sample.

Regarding educational level, 20.0% (31/152) of participants were unskilled, while the majority had completed secondary education (59.0%; 90/152), with 9.0% (14/152) having a post-secondary degree and 11.0% (17/152) having completed university-level education.

A total of 26.0% (39/152) of patients reported owning a household farm. Consumption of processed meat products was reported by 45.0% (68/152) of participants, and 23.0% (35/152) were involved in the production of such products. Consumption of undercooked meat was documented in 15.0% (23/152) of patients, while 11.0% (16/152) reported occupational contact with animals.

Most participants obtained drinking water from a public supply system (84.0%; 128/152), while 9.0% (13/152) used well water, and 5.0% (8/152) used bottled water. Sanitary conditions also varied: 52.0% (79/152) of participants reported using public sewage systems, 46.0% (70/152) used septic tanks, and 2.0% (3/152) had pit latrines.

At baseline, 20.4% (31/152) were anti-HEV IgG-positive. At 6 and 12 months post-transplant, seroprevalence decreased to 19% (29/152) and 16.4% (25/152), respectively (Table 3).

Patients were stratified into three subgroups based on their serological status before transplantation to assess the dynamics of anti-HEV IgG antibodies over time:

1. Seronegative patients (75.6%; 115/152)—Remained seronegative throughout follow-up.
2. Persistently seropositive patients (20.4%; 31/152)—Patients were anti-HEV IgG-positive pre-transplant, with seroprevalence declining from 20.4% at baseline to 19.0% at 6 months and 16.4% at 12 months.
3. IgG seroconverters (3.9%; 6/152)—These patients were initially seronegative but developed anti-HEV IgG positivity after transplantation. Four patients seroconverted within the first 6 months and two between the 6th and 12th months post-transplant. All tested negative for HEV RNA. Among them, four were male, and three had alcohol-related liver disease. Mildly elevated liver enzymes were noted in three patients, and one patient showed signs of mild acute cellular rejection. Immunosuppressive regimens included cyclosporine in four cases and tacrolimus in two (Table 4).

## 4. Discussion

This study offers an extensive analysis of HEV exposure in LT recipients in Croatia. We observed a notable anti-HEV IgG seroprevalence of 19.8%, indicating substantial prior exposure, with a very low rate of seroconversion post-transplant and no cases of acute or chronic HEV infection. Collectively, these findings suggest that while LT recipients in Croatia are exposed to HEV, they are not at heightened risk of developing chronic disease.

The 19.8% anti-HEV IgG seroprevalence among LT recipients closely resembles the rate in the Croatian blood donor population (21.5%) [13], reinforcing that prior HEV exposure is common in the general population. Compared to other European cohorts, Croatian transplant recipients show higher seroprevalence than those in Spain (7.4%) [14], the Netherlands (2.1%) [5], and Greece (1.3%) [4] but comparable rates to Italy (19.2–33%) [15,16] and Germany (28.8%) [6]. Japan, by contrast, reports much lower prevalence (2.9%) [17], whereas southwestern France, a known hyperendemic region, records 29% [7]. These international comparisons position Croatia among countries with a moderately high HEV exposure burden in immunosuppressed patients.

Despite the elevated seroprevalence, no HEV RNA was detected in any of the 1070 post-transplant samples, regardless of collection time point. This suggests that HEV infection, while likely frequent, is mostly subclinical and self-limiting in this population, and does not progress to chronic infection. Such findings support a reassuring clinical outlook for LT recipients in this setting.

International variability in HEV seroprevalence may reflect differences in assay performance, regional dietary practices (e.g., pork consumption), sanitation standards, and HEV prevalence in animal reservoirs. In our cohort, older age was a consistent independent predictor of seropositivity, which supports the theory of cumulative lifetime exposure [7,18]. Gender was not associated with HEV exposure, mirroring previous studies [13].

Given that domestic pigs are the main HEV reservoir and that home-produced food is widely consumed in Croatia, it is notable that rural residency and consumption of home-cured meat were not associated with seropositivity. Over 60% of participants consumed these products, and 20% reported avoiding undercooked meat. The absence of a significant association may be partly explained by recall bias, socially desirable reporting, or changes in dietary habits after transplantation, which could obscure true pre-transplant exposure patterns.

Drinking water source and sanitation (municipal vs. well; sewer vs. septic tank) also showed no association. On the other hand, non-tertiary level of education was significantly linked to seropositivity, likely serving as a proxy for lower health literacy, hygiene standards, and awareness [17].

HEV circulation in pigs has been documented in Croatia since 2010 [8,9], with animal seroprevalence reaching over 60%, especially during peak shedding periods around the third month of life [19]. Nevertheless, pig exposure variables did not correlate with human seropositivity, which may suggest that other routes of transmission, possibly blood transfusion or environmental contamination—merit further investigation.

Assay performance remains a critical issue in comparing HEV seroprevalence data across studies. Commercial ELISA kits vary widely in sensitivity and specificity, often producing divergent results. In the UK, estimates ranged from 3.6% to 16.2% depending on the test used, with sensitivity from 56% to 98% [20]. Our study employed the RecomWell ELISA (Mikrogen), validated in Croatian donor studies, which consistently shows seroprevalence between 17.8% and 20% [13].

We tracked anti-HEV IgG seropositivity at three intervals: pre-transplant (20.3%), six months post-transplant (19%), and twelve months post-transplant (16.4%). The gradual decline may be explained by waning immunity due to immunosuppressive therapy [21], and by the limited sensitivity of serological assays in immunocompromised patients [22], rather than true loss of immunity.

A small proportion (3.9%) of initially seronegative LT recipients seroconverted during follow-up without concomitant HEV RNA detection. These events cannot be classified as confirmed previous infections because (i) RNA negativity and the absence of anti-HEV IgM testing preclude establishing timing/acuity; (ii) passive transfer of anti-HEV antibodies via transfused blood products remains plausible post-transplant; (iii) donor HEV serology/RNA were unavailable; and (iv) given infrequent sampling and batch testing, transient viremia cannot be excluded. Although this proportion exceeds some prior reports in transplant populations (1–2.1%) [5,23] and aligns with observations from southwestern France [7], we interpret these as serologic kinetics of uncertain clinical significance, rather than definitive evidence of new HEV infection.

Interestingly, no cases of chronic HEV infection were identified. Immunosuppressive regimens may influence viral persistence—tacrolimus, for instance, has been linked to increased HEV replication and chronic infection risk [24], while cyclosporine, the predominant immunosuppressant used in this cohort, may limit viral replication (at least in hepatitis C) [25]. However, its impact on HEV remains unclear and warrants dedicated study.

This study has several limitations. The primarily cross-sectional design and routine sampling schedule may have missed transient or asymptomatic infections. In the prospective cohort, samples were collected at only three time points, stored, and tested at the end of this study rather than in real time, which may have resulted in missed short-lived viremia, delayed seroconversion, or imprecise timing of infection.

Anti-HEV IgM testing was not performed, and donor HEV serology/RNA were not assessed, limiting confirmation of acuity and evaluation of donor-derived transmission. Assay-related limitations, including lower sensitivity in immunosuppressed patients, may have led to underestimation of true prevalence; although RNA testing was performed at each time point, very brief periods of detectable viremia could still have been missed. Accordingly, IgG seroconversion without concomitant viremia cannot be interpreted as confirmed infection and may reflect passive antibody transfer from transfusions. We did not present univariable screens or apply multiplicity corrections; primary inference relied on a prespecified multivariable model (ORs, 95% CIs), acknowledging a residual risk of chance associations. Finally, the 12-month follow-up and rarity of events may have missed late-onset chronic infection and limited power for adjusted incidence analyses, and the single-center design may constrain generalizability.

## 5. Conclusions

Our study highlights that prior exposure to HEV is frequent among LT recipients in Croatia, with a seroprevalence rate comparable to the general population. However, active infection—especially chronic HEV—is exceptionally rare in this cohort.

Key risk factors for HEV seropositivity include older age and non-tertiary level of education, suggesting the role of cumulative exposure and health literacy in infection risk. In contrast, dietary habits, environmental conditions, and direct contact with HEV animal reservoirs showed no significant association.

Importantly, the lack of chronic HEV infections, even in an immunosuppressed population, supports a reassuring clinical outlook. Nonetheless, the potential for asymptomatic and transient infections, as well as the limitations of current serological assays, reinforces the need for continued epidemiological surveillance, enhanced risk stratification tools, and evaluation of preventive measures, including vaccination and donor screening.

Future research should explore transmission pathways beyond dietary and rural exposure and clarify the role of immunosuppression in HEV dynamics.

## Figures and Tables

**Table 1:** Characteristics of liver transplant recipients and prevalence of anti-HEV IgG antibodies, Croatia (n = 766).

Parameter	Total (n = 766)	Anti-HEV IgG Negative	Anti-HEV IgG Positive	*p*-Value
Age at transplantation, median years (IQR 25th–75th percentile)	56 (18–80)	55 (49–62)	56 (50.5–62.5)	0.122
Male sex, n (%)	532 (68.6%)	423 (68.9%)	109 (71.7%)	0.499
Age at sampling, median years (IQR)	60 (18–80)	59 (53–65)	61 (56–66)	0.023
HEV RNA positive, n (%)	0	0	0	—
Etiology of liver disease:				
- Alcohol related liver disease, n (%)	360 (47.0%)	291 (47.5%)	69 (45.4%)	0.651
- Viral hepatitis B and C, n (%)	131 (17.1%)	107 (17.5%)	24 (15.8%)	0.635
- Primary liver tumors, n (%)	199 (26.0%)	154 (25.1%)	45 (29.6%)	0.259
No. immunosuppressive regimen				
- Monotherapy, n (%)	46 (6.0%)	39 (6.0%)	7 (5.0%)	0.46
- Dual therapy, n (%)	593 (77.4%)	478 (78.0%)	115 (76.0%)	
- Triple therapy, n (%)	43 (5.6%)	32 (5.0%)	11 (7.0%)	
- Missing data, n (%)	84 (11.0%)			
Type of calcineurin inhibitor				
Tacrolimus, n (%)	409 (53.4%)	325 (53.0%)	84 (55.0%)	0.30
Cyclosporine, n (%)	325 (42.4%)	262 (43.0%)	63 (41.0%)	
Other, n (%)	9 (1.2%)	9 (1.0%)	0	
Missing data, n (%)	23 (3.0%)			
Laboratory parameters:				
- Leukocytes (×10^9^/L), median (IQR)	6.25 (1.83–33.35)	6.24 (4.88–7.67)	6.33 (4.96–7.86)	0.549
- Lymphocytes (×10^9^/L), median (IQR)	1.62 (0.36–11.21)	1.63 (1.2–2.14)	1.56 (1.23–2.17)	0.812
- Platelets (×10^9^/L), median (IQR)	168.00 (29–758)	167 (131–214)	174.5 (137–208.5)	0.239
- Creatinine (µmol/L), median (IQR)	89.00 (5.5–710)	88 (72–109)	90 (77.5–109.5)	0.197
- Total bilirubin (µmol/L), median (IQR)	15.00 (3–253)	15 (12–22)	15 (11–20)	0.550
- AST (U/L), median (IQR)	29.00 (9–994)	29 (22–41)	30 (23–41)	0.506
- ALT (U/L), median (IQR)	28.00 (5–808)	28 (18–45)	27 (17.5–40)	0.626
Level of education:				
- No formal education, n (%)	145 (19.5%)	112 (19.0%)	33 (21.7%)	0.017
- Secondary school, n (%)	445 (61.2%)	353 (9.7%)	92 (60.5%)	
- Post-secondary non-tertiary education, n (%)	71 (9.5%)	51 (8.6%)	20 (13.2%)	
- Tertiary education, n (%)	82 (11.03%)	75 (12.7%)	7 (4.6%)	
- Missing data, n (%)	23 (3.0%)			
Place of residence:				
- Urban, n (%)	387 (52.1%)	314 (53.1%)	73 (48.0%)	0.261
- Rural, n (%)	356 (47.9%)	277 (46.9%)	79 (52.0%)	
- Missing data, n (%)	23 (3.0%)			
Number of household members:				
- <2, n (%)	134 (18.1%)	114 (19.4%)	20 (13.2%)	0.118
- 3–4, n (%)	483 (65.2%)	372 (63.2%)	111 (73.0%)	
- >5, n (%)	124 (16.7%)	103 (17.5%)	21 (13.8%)	
- Missing data, n (%)	25 (3.3%)			
Own livestock, n (%)	186 (24.3%)	146 (24.7%)	40 (26.3%)	0.628
Vegetarian, n (%)	4 (0.5%)	2 (0.3%)	2 (1.3%)	0.142
Consume cured meat, n (%)	489 (63.8%)	391 (66.2%)	98 (64.9%)	0.771
Produce cured meat, n (%)	171 (22.3%)	141 (23.9%)	30 (19.7%)	0.282
Consume undercooked meat, n (%)	154 (20.1%)	122 (20.6%)	32 (21.1%)	0.912
Work with animals, n (%)	113 (14.8%)	84 (14.2%)	29 (19.1%)	0.136
Drinking water- public supply, n (%)	602 (86.9%)	479 (81.5%)	123 (80.9%)	0.879
- Missing data, n (%)	73 (9.5%)			
Sanitary facilities:				
- Public sewer, n (%)	430 (58.2%)	348 (59.1%)	83 (54.6%)	0.602
- Septic tank, n (%)	304 (41.2%)	238 (40.4%)	68 (44.7%)	
- Outdoor toilet, n (%)	4 (0.54%)	3 (0.5%)	1 (0.7%)	
- Missing data, n (%)	28 (3.7%)			
Blood transfusion after LT, n (%)	498 (65.4%)	393 (64.4%)	105 (69.1%)	0.24
- Missing data, n (%)	28 (3.7%)			
Recent travel abroad (past 3 years), n (%)	213 (27.8%)	174 (29.6%)	39 (25.7%)	0.340

Values are n (%). Percentages use available denominators (excluding missing).

**Table 2:** Multivariable logistic regression analysis of factors associated with anti-HEV IgG seropositivity in liver transplant recipients.

Variable	B	Wald	*p*-Value	OR (95% CI)
Age at time of sampling	0.01	6.8	0.009	1.03 (1.01–1.05)
Tertiary education				
–No	Ref.	7.27	0.007	0.33 (0.15–0.74)
–Yes	0.41			
>2 household members				
–No	Ref.	2.95	0.086	1.59 (0.94–2.71)
–Yes	0.27			
Vegetarian diet				
–No	Ref.	3.73	0.053	7.66 (0.97–60.44
–Yes	1.05			
Occupational exposure to animals				
–No	Ref.	0.93	0.334	1.26 (0.79–2.04)
–Yes	0.24			
Chronic hepatitis B				
–No	Ref.	2.64	0.105	1.87 (0.88–3.99)
–Yes	0.39			
Chronic hepatitis C				
–No	Ref.	1.99	0.158	0.65 (0.35–1.18)
–Yes	0.31			

Multivariable logistic regression model including variables with *p* < 0.20 in preliminary bivariate comparisons. B = regression coefficient; Wald = Wald χ^2^ statistic; OR = odds ratio; CI = confidence interval; Ref. = reference category.

**Table 3:** Baseline characteristics of the prospective cohort.

Parameter	Value (n = 152)
Median age at sampling (years), IQR	59.0 (23–76)
Male gender, n (%)	66.0% (100)
Median age at LT (years)	59.5
Etiology of liver disease	
- Alcohol related liver disease, n (%)	56 (36.8%)
- Autoimmune liver diseases, n (%)	19 (12.5%)
- Primary liver tumors, n (%)	45 (29.6%)
Type of calcineurin inhibitor	
- Tacrolimus, n (%)	106 (69.7%)
- Cyclosporine, n (%)	46 (30.3%)
Serological and molecular results	
- Anti-HEV IgG positive before LT*, n (%)	31 (20%)
- Anti-HEV IgG positive 6 months after LT*, n (%)	29 (19%)
- Anti-HEV IgG positive 12 months after LT*, n (%)	25 (16.4%)
- HEV RNA positive before LT*, n (%)	0 (0%)
- HEV RNA positive 6 months after LT*, n (%)	0 (0%)
- HEV RNA positive 12 months after LT*, n (%)	0 (0%)
Laboratory data	
- Leukocytes (×10^9^/L), median (IQR)	5.49 (1.16–15.85)
- Lymphocytes (×10^9^/L), median (IQR)	1.3 (0.09–6.71)
- Platelets (×10^9^/L), median (IQR)	106 (15–789)
- Creatinine (µmol/L), median (IQR)	68 (38–649)
- Total bilirubin (µmol/L), median (IQR)	35.5 (5–840)
AST (U/L), median (IQR)	56.5 (17–1395)
ALT (U/L), median (IQR)	37 (11–1340)
Education level	
- No formal education, n (%)	31 (20%)
- Secondary school, n (%)	90 (59%)
- Post-secondary non-tertiary education, n (%)	14 (9%)
- Tertiary education, n (%)	17 (11%)
Place of residence	
- Urban, n (%)	77 (51%)
- Rural, n (%)	75 (49%)
Household Size	
- <2 members, n (%)	31 (20%)
- 3–4 members, n (%)	90 (59%)
- >5 members, n (%)	31 (20%)
Exposure and lifestyle factors	
- Owns domestic livestock, n (%)	39 (26%)
- Vegetarian, n (%)	1 (1%)
- Consumes cured meat products, n (%)	68 (45%)
- Produces cured meat products, n (%)	35 (23%)
- Consumes undercooked meat, n (%)	23 (15%)
- Works with animals, n (%)	16 (11%)
Drinking water source	
- Public supply, n (%)	128 (84%)
- Cistern, n (%)	0 (0%)
- Well, n (%)	13 (9%)
- Bottled	8 (5%)
- Spring	3 (2%)
Sanitation facilities	
- Public sewer	79 (52%)
- Septic tank	70 (46%)
- Pit latrine	3 (2%)
- Other	
Blood transfusion after LT*	65 (43%)
Travel abroad in last 3 years	42 (28%)

LT* = Liver transplantation.

**Table 4:** Clinical and epidemiological characteristics of HEV seroconverted liver transplant patients.

Parameter	Patient 1	Patient 2	Patient 3	Patient 4	Patient 5	Patient 6
Year of birth	1950	1959	1958	1944	1947	1982
Gender	Male	Male	Male	Female	Male	Female
Anti-HEV IgG pre-transplant	Neg	Neg	Neg	Neg	Neg	Neg
Anti-HEV IgG at 6 months	Pos	Pos	Pos	Pos	Neg	Neg
Anti-HEV IgG at 12 months	Pos	Pos	Neg	Neg	Pos	Pos
Underlying liver disease	HCC ^1^	HCC ^1^, HCV ^2^	HCC ^1^, HCV ^2^	ALD ^3^	HCC ^1^, HBV ^4^	PBC ^5^
Education level	Secondary	Secondary	Secondary	Secondary	Secondary	Tertiary
No. of immunosuppressive drugs	3	2	2	2	2	2
CNI ^6^ drug	TAC ^7^	CYC ^8^	CYC ^8^	TAC ^7^	CYC ^8^	CYC ^8^
CNI ^6^ concentration at 6 months	9	81	39	7.1	204	300
CNI ^6^ concentration at 12 months	4	134	91	6.3	155	225
Leukocytes (×10^9^/L)	6.22	9.15	3.95	8.3	8.55	4.89
Lymphocytes (×10^9^/L)	0.98	1.51	0.92	1.49	1.33	1.45
Platelets (×10^9^/L)	172	195	76	360	307	133
Total bilirubin (µmol/L)	23	8	17	11	15	22
Creatinine (µmol/L)	88	73	75	66	85	101
AST (U/L)	22	31	26	40	44	39
ALT (U/L)	31	29	16	19	22	63
Leukocytes at 12 months (×10^9^/L)	3.12	9.61	4.15	7.78	6.52	4.02
Lymphocytes at 12 months (×10^9^/L)	1.12	1.7	1.77	1.43	1.66	1.51
Platelets at 12 months (×10^9^/L)	149	196	87	284	177	128
Bilirubin at 12 months (µmol/L)	23	9	14	16	16	18
Creatinine at 12 months (µmol/L)	87	77	78	41	84	93
AST at 12 months (U/L)	20	25	27	41	30	40
ALT at 12 months (U/L)	24	29	28	21	26	43
Number of household members	3	3	2	2	4	3
Place of residence	Urban	Urban	Urban	Urban	Rural	Urban
Owns livestock	No	No	No	No	No	No
Vegetarian	No	No	No	No	No	No
Consumption of cured meat	No	No	No	Yes	Yes	No
Homemade cured meat production	No	No	No	No	No	No
Consumption of undercooked meat	No	No	No	No	No	Yes
Work related to animals	No	No	No	No	No	No
Drinking water source	Public	Public	Public	Public	Public	Bottled
Sanitary facility	Sewer	Sewer	Sewer	Sewer	Septic tank	Sewer
Traveling abroad (last 3 years)	No	No	Yes	Yes	No	Yes

HCC ^1^ = Hepatocellular carcinoma; HCV ^2^ = Hepatitis C virus; ALD ^3^ = Alcohol related liver disease; HBV ^4^ = Hepatitis B virus; PBC ^5^ = Primary biliary cholangitis; CNI ^6^ = Calcineurin inhibitor; TAC ^7^ = Tacrolimus; CYC ^8^ = Cyclosporine.

## Data Availability

The raw data supporting the conclusions of this article will be made available by the corresponding authors on reasonable request.

## Abbreviations

The following abbreviations are used in this manuscript:

HEV	Hepatitis E virus
LT	Liver transplant
IgG	Immunoglobulin G
RNA	Ribonucleic acid
PCR	Polymerase chain reaction
CNI	Calcineurin inhibitor
AST	Aspartate aminotransferase
ALT	Alanine aminotransferase
CRP	C-reactive protein
RT-qPCR	Reverse transcription quantitative polymerase chain reaction
ORF3	Open reading frame 3
Ct	Cycle threshold
SPSS	Statistical Package for the Social Sciences
ANOVA	Analysis of variance
BMI	Body mass index
ELISA	Enzyme-linked immunosorbent assay
SD	Standard deviation
CI	Confidence interval
OR	Odds ratio
CVD	Cardiovascular disease
HCV	Hepatitis C virus
HCV	Hepatitis E virus
HCC	Hepatocellular carcinoma
HBV	Hepatitis B virus
TAC	Tacrolimus
CYC	Cyclosporine
ALD	Alcohol-related liver disease
PBC	Primary biliary cholangitis

## References

[1] Goel A., Aggarwal R. (2016). Advances in hepatitis E-II: Epidemiology, clinical manifestations, treatment and prevention. Expert Rev. Gastroenterol. Hepatol..

[2] Colson P., Borentain P., Queyriaux B., Kaba M., Moal V., Gallian P., Heyries L., Raoult D., Gerolami R. (2010). Pig liver sausage as a source of hepatitis E virus transmission to humans. J. Infect. Dis..

[3] Kamar N., Selves J., Mansuy J.M., Ouezzani L., Péron J.M., Guitard J., Cointault O., Esposito L., Abravanel F., Danjoux M. (2008). Hepatitis E virus and chronic hepatitis in organ-transplant recipients. N. Engl. J. Med..

[4] Sinakos E., Gioula G., Liava C., Papa A., Papadopoulou E., Tsakni E., Fouzas I., Akriviadis E. (2018). Prevalence of hepatitis E in liver transplant recipients in Greece. Epidemiol. Infect..

[5] Haagsma E.B., Niesters H.G., van den Berg A.P., Riezebos-Brilman A., Porte R.J., Vennema H., Reimerink J.H.J., Koopmans M.P.G. (2009). Prevalence of hepatitis E virus infection in liver transplant recipients. Liver Transpl..

[6] Darstein F., Häuser F., Mittler J., Zimmermann A., Lautem A., Hoppe-Lotichius M., Otto G., Lang H., Galle P., Zimmermann T. (2020). Hepatitis E Is a Rare Finding in Liver Transplant Patients With Chronic Elevated Liver Enzymes and Biopsy-Proven Acute Rejection. Transplant Proc..

[7] Buffaz C., Scholtes C., Dron A.G., Chevallier-Queyron P., Ritter J., André P., Ramière C. (2014). Hepatitis E in liver transplant recipients in the Rhône-Alpes region in France. Eur. J. Clin. Microbiol. Infect. Dis..

[8] Prpić J., Černi S., Škorić D., Keros T., Brnić D., Cvetnić Ž., Jemeršić L. (2015). Distribution and Molecular Characterization of Hepatitis E virus in Domestic Animals and Wildlife in Croatia. Food Environ. Virol..

[9] Jemeršić L., Prpić J., Brnić D., Keros T., Pandak N., Đaković Rode O. (2019). Genetic diversity of hepatitis E virus (HEV) strains derived from humans, swine and wild boars in Croatia from 2010 to 2017. BMC Infect. Dis..

[10] Mrzljak A., Jemersic L., Savic V., Balen I., Ilic M., Jurekovic Z., Pavicic-Saric J., Mikulic D., Vilibic-Cavlek T. (2021). Hepatitis E Virus in Croatia in the "One-Health" Context. Pathogens.

[11] Mrzljak A., Dinjar-Kujundzic P., Vilibic-Cavlek T., Jemersic L., Prpic J., Dakovic-Rode O., Kolaric B., Vince A. (2019). Hepatitis E seroprevalence and associated risk factors in Croatian liver transplant recipients. Rev. Soc. Bras. Med. Trop..

[12] Jothikumar N., Cromeans T.L., Robertson B.H., Meng X.J., Hill V.R. (2006). A broadly reactive one-step real-time RT-PCR assay for rapid and sensitive detection of hepatitis E virus. J. Virol. Methods.

[13] Miletić M., Vuk T., Hećimović A., Stojić Vidović M., Jemeršić L., Jukić I. (2019). Estimation of the hepatitis E assay-dependent seroprevalence among Croatian blood donors. Transfus. Clin. Biol..

[14] Riveiro-Barciela M., Buti M., Homs M., Campos-Varela I., Cantarell C., Crespo M., Castells L., Tabernero D., Quer J., Esteban R. (2014). Cirrhosis, liver transplantation and HIV infection are risk factors associated with hepatitis E virus infection. PLoS ONE.

[15] Binda B., Picchi G., Bruni R., Di Gasbarro A., Madonna E., Villano U., Pisani G., Carocci A., Marcantonio C., Montali F. (2025). The Prevalence, Risk Factors, and Outcomes of Hepatitis E Virus Infection in Solid Organ Transplant Recipients in a Highly Endemic Area of Italy. Viruses.

[16] Zanotto E., Rittà M., Pittaluga F., Martini S., Ciotti M., Cavallo R., Costa C. (2023). Seroprevalence of hepatitis E virus in liver transplant patients in Turin, Italy. Panminerva Med..

[17] Inagaki Y., Oshiro Y., Tanaka T., Yoshizumi T., Okajima H., Ishiyama K., Nakanishi C., Hidaka M., Wada H., Hibi T. (2015). A nationwide survey of hepatitis E virus infection and chronic hepatitis E in liver transplant recipients in Japan. EBioMedicine.

[18] European Association for the Study of the Liver (2018). EASL Clinical Practice Guidelines on hepatitis E virus infection. J. Hepatol..

[19] Dremsek P., Joel S., Baechlein C., Pavio N., Schielke A., Ziller M., Dürrwald R., Renner C., Groschup M.H., Johne R. (2013). Hepatitis E virus seroprevalence of domestic pigs in Germany determined by a novel in-house and two reference ELISAs. J. Virol. Methods.

[20] Bendall R., Ellis V., Ijaz S., Ali R., Dalton H. (2010). A comparison of two commercially available anti-HEV IgG kits and a re-evaluation of anti-HEV IgG seroprevalence data in developed countries. J. Med. Virol..

[21] Zhang J., Zhang X.F., Zhou C., Wang Z.Z., Huang S.J., Yao X., Liang Z.-L., Wu T., Li J.-X., Yan Q. (2014). Protection against hepatitis E virus infection by naturally acquired and vaccine-induced immunity. Clin. Microbiol. Infect..

[22] Rossi-Tamisier M., Moal V., Gerolami R., Colson P. (2013). Discrepancy between anti-hepatitis E virus immunoglobulin G prevalence assessed by two assays in kidney and liver transplant recipients. J. Clin. Virol..

[23] Abravanel F., Lhomme S., Chapuy-Regaud S., Mansuy J.M., Muscari F., Sallusto F., Rostaing L., Kamar N., Izopet J. (2014). Hepatitis E virus reinfections in solid-organ-transplant recipients can evolve into chronic infections. J. Infect. Dis..

[24] Kamar N., Garrouste C., Haagsma E.B., Garrigue V., Pischke S., Chauvet C., Dumortier J., Cannesson A., Cassuto-Viguier E., Thervet E. (2011). Factors associated with chronic hepatitis in patients with hepatitis E virus infection who have received solid organ transplants. Gastroenterology.

[25] Ciesek S., Steinmann E., Wedemeyer H., Manns M.P., Neyts J., Tautz N., Madan V., Bartenschlager R., von Hahn T., Pietschmann T. (2009). Cyclosporine A inhibits hepatitis C virus nonstructural protein 2 through cyclophilin A. Hepatology.

